# Nephrons, podocytes and chronic kidney disease: Strategic antihypertensive therapy for renoprotection

**DOI:** 10.1038/s41440-022-01061-5

**Published:** 2022-10-12

**Authors:** Kotaro Haruhara, Go Kanzaki, Nobuo Tsuboi

**Affiliations:** Division of Nephrology and Hypertension, Department of Internal Medicine, The Jikei University School of Medicine, Tokyo, Japan

**Keywords:** Chronic kidney disease, Hypertension, Nephron, Podocyte, Salt handling

## Abstract

Chronic kidney disease (CKD) is one of the strongest risk factors for hypertension, and hypertension can exacerbate the progression of CKD. Thus, the management of CKD and antihypertensive therapy are inextricably linked. Research over the past decades has shown that the human kidney is more diverse than initially thought. Subjects with low nephron endowment are at increased risk of developing CKD and hypertension, which is consistent with the theory of the developmental origins of health and disease. Combined with other lifetime risks of CKD, hypertension may lead to a vicious cycle consisting of podocyte injury, glomerulosclerosis and further loss of nephrons. Of note, recent studies have shown that the number of nephrons correlates well with the number of podocytes, suggesting that these two components are intrinsically linked and may influence each other. Both nephrons and podocytes have no or very limited regenerative capacity and are destined to decrease throughout life. Therefore, one of the best strategies to slow the progression of CKD is to maintain the “numbers” of these essential components necessary to preserve renal function. To this end, both the achievement of an optimal blood pressure and a maximum reduction in urinary protein excretion are essential. Lifestyle modifications and antihypertensive drug therapy must be carefully individualized to address the potential diversity of the kidneys.

## Graphical abstract

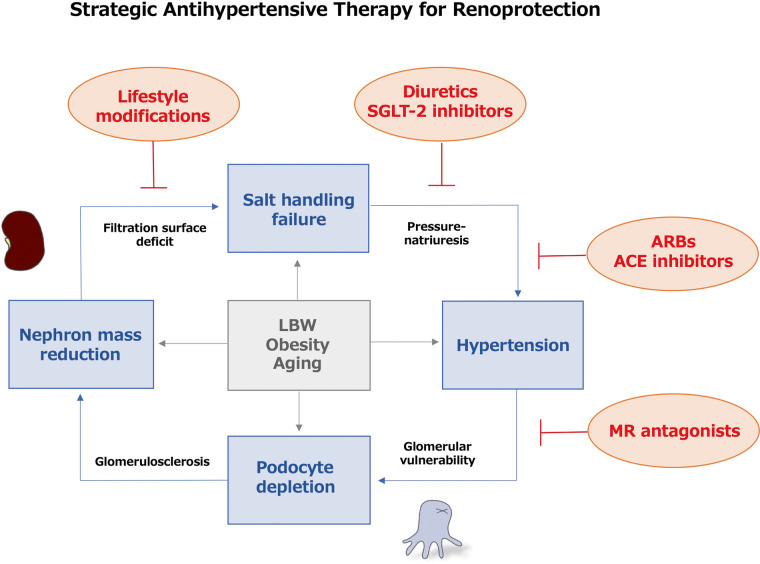

## Introduction

The human kidney is composed of functional units called nephrons [1]. Numerous epidemiological studies have demonstrated that a low birth weight, a known surrogate marker for a low nephron number, is associated with an increased incidence of hypertension and kidney disease in later life [2]. These findings are consistent with the currently established theory of the developmental origins of health and disease, which proposes a close relationship between an impaired gestational environment and the risk of developing noncommunicable diseases in adulthood [3]. However, because hypertension is multifactorial and complex, the causal relationship between the nephron number and the development of hypertension remains controversial [4].

From a renal histopathologic perspective, chronic kidney disease (CKD) can be viewed as a comprehensive syndrome in which nephrons are gradually lost and the remaining nephrons show a transition to compensatory failure. Glomerular podocytes are largely responsible for the maintenance of glomerular structure and filtration function [5]. Depending on the number and function of the remaining nephrons and podocytes in the glomerulus, hemodynamic loading on each glomerulus can induce filtration dysfunction and persistent leakage of serum protein into the urine (proteinuria), causing protein overload injury throughout the kidney [6]. In many cases, the severity of podocyte injury correlates with the total amount of urinary protein excretion [7, 8]. Therefore, treatment of CKD should be focused on protecting podocytes from injury and minimizing urinary protein excretion.

To better understand the complex pathogenesis of CKD and hypertension, an understanding of the structural differences in the kidney that underlie the various clinical manifestations is essential. The primary objective of this review is to summarize the current knowledge concerning the number of nephrons and podocytes in relation to CKD and hypertension. Effective strategic approaches to CKD patients are then discussed, with a focus on antihypertensive therapy for renoprotection.

Point of view **Clinical relevance**

Recent human and animal studies have shown that congenital and/or acquired reductions in nephrons and podocytes are important pathophysiological components in the common pathway of CKD progression. Combined lifestyle modifications and antihypertensive therapy are important to halt the progressive loss of these vital components for kidney health.

**Future direction**

For effective population-level intervention for CKD, a noninvasive and simple method of evaluating the structural diversity in the kidneys, including the numbers of nephrons and podocytes, is needed.

**Considerations for the Asian population**

There is substantial concern that the number of nephrons and podocytes in the Asian population has been declining in recent years. A public health approach is needed to educate the population on the importance of proper dietary habits and a maternal-fetal environment suitable for kidney maturation.

## Nephron number, hypertension, and CKD progression

### Variation in nephron number in humans

**Fig. 1:**
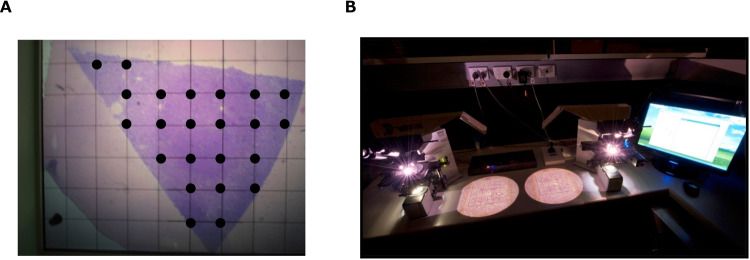
Estimating the total nephron number in humans using the physical disector/fractionator combination. **A** The area of the section is estimated using point counting. **B** Glomeruli are counted using physical dissectors. A known fraction of the area of the sections is used for glomerular counting. Then, simple algebra is used to estimate the number of glomeruli in the whole kidney. Photographs were taken at the Department of Anatomy and Developmental Biology, Monash University

Most medical textbooks state that there are one million nephrons per kidney. However, even in healthy kidneys, there are individual differences in the weight, cortical volume, and number of nephrons, with large variations [9]. In recent years, an unbiased stereological method, namely, the physical disector/fractionator combination, for estimating total glomerular number in the kidneys has been developed [10] (Fig. 1). This method identified greater individual differences in nephron number in healthy people without kidney disease than expected, with a maximum difference of approximately 13-fold [11]. Furthermore, such differences in nephron number have been associated with the incidence and pathogenesis of hypertension [12]. As the nephron number in humans is determined by the late fetal period (36 weeks) and does not increase thereafter, the number between individuals has become regarded as a “potential difference” in renal functional reserve [13].

Previous studies have identified birth weight, sex, race, and genetic factors as determinants of the number of nephrons in an individual, with the maternal-fetal environment being considered to have a strong influence on the determination of nephron number [14]. A representative report demonstrating such a finding involved an evaluation of the nephron numbers in the Aboriginal population of Australia. It was noted that Aboriginal people have an average of 700,000 nephrons per kidney and have a higher incidence of low birth weight and end-stage kidney disease (ESKD) than non-Aboriginal Australians [15].

Following these Aboriginal nephron number studies, 6 other races (Danish, German, American, Australian, and Senegalese) have been evaluated thus far, and human nephron numbers have been reported to range from 210,000 to 2.7 million [12, 16–19]. However, there have been no reports on Asians, including Japanese, so we measured nephron numbers in adult Japanese subjects with no apparent primary kidney diseases. The comparison of autopsy kidneys from 9 age- and sex-matched normotensive, 9 hypertensive, and 9 CKD subjects showed that the total nephron number (total nonsclerotic glomeruli) in the normotensive group was approximately 670,000 per kidney, whereas the hypertensive group had a lower number of approximately 420,000 per kidney [20]. This study showed that the nephron number of the Japanese in the normotensive group was similar to that of Aboriginal Australians and ranked in the lowest group among the races. This is an important finding that suggests an association between the low nephron number and high prevalence of hypertension and CKD in Japan [21].

### Maternal-fetal nutrition as a factor related to nephron number and the development of hypertension

Various studies have identified birth weight, race, body size, and sex as factors that contribute to nephron numbers. The maternal-fetal environment has attracted attention as one of the most important factors [2]. Nephrogenesis in humans is completed by the 36th week of gestation, and no further nephrons are developed after birth. A positive correlation between birth weight and total nephron number has also been observed, suggesting a significant influence of the maternal-fetal environment on the development of nephrons [22]. Factors that influence nephron formation include maternal nutritional status, micronutrients, smoking and alcohol habits, drug use, and maternal renal dysfunction, and the molecular mechanisms have been elucidated from animal model studies [23].

Blood pressure in childhood is inversely related to birth weight. Law et al. reported that the relationship between blood pressure and birth weight at 4 years old was characterized by systolic blood pressure being higher in cases with a lower birth weight and higher placental weight and that systolic blood pressure at 4 years old was on average 2.6 mmHg higher in cases with a birth weight of ≤ 3000 g than in those with a birth weight of > 3600 g [24]. Several reports have indicated that blood pressure is inversely correlated with birth weight, even in adolescents approximately 20 years old [25]. Gennser et al. also reported a significant inverse association between birth weight and diastolic blood pressure in adults [26]. A low birth weight had an odds ratio of 3.6 for the development of elevated diastolic blood pressure.

As maternal and childhood height were also inversely correlated with the incidence of cardiovascular diseases, Barker et al. hypothesized that there would be an association between *in utero* growth and adult blood pressure [3]. An inverse association existed between the height of the body at birth and systolic blood pressure, independent of the weight of the adult body and gestational age at birth. For systolic blood pressure at 46–54 years old, the blood pressure decreased by an average of 11 mmHg as birth weight increased from < 5.5 lbs to > 7.5 lbs. The decrease in birth weight and increase in placental weight had independent effects on the increases in blood pressure in adulthood, with the most marked increases in blood pressure being noted in cases with a low birth weight and large placental weight. Furthermore, Barker et al. reported that the risk of death from ischemic heart disease was inversely related to birth weight and weight at one year of age [27]. It is clear that low birth weight is associated with the development of postnatal hypertension and that the effect of increased blood pressure is amplified with increasing age. A 1 kg decrease in birth weight was shown to be associated with an increase in systolic blood pressure of 1–3 mmHg in adolescents and an increase of 5.2 mmHg at 64–71 years old.

Reduced nephron numbers are associated with a variety of abnormalities during embryonic life. It is known that continuous low protein feeding of the mother results in a reduction in fetal birth weight, with a proportionally smaller kidney and reduced nephron number. Furthermore, glomerulosclerotic lesions develop in fetal rats as they mature after birth, and blood pressure increases in a salt-sensitive manner. Although evidence in humans is lacking, in epidemiological studies, historical events such as the Dutch Winter Famine, the Siege of Leningrad, and the Great Leap Forward in China have shown links between malnutrition during pregnancy and the subsequent development of impaired glucose tolerance, obesity, hypertension, and mental illness.

Given the above information, Brenner et al. proposed the hypothesis that low-birth-weight infants are born with impaired renal development resulting in a reduced nephron number, which in turn leads to systemic and glomerular hypertension, forming a vicious cycle that gradually progresses to the eventual development of hypertension as an adult. They further hypothesized that a reduced nephron number and total glomerular filtration surface area were associated with the development of hypertension.

### Nephron numbers in autopsy studies and the development of hypertension

The association between essential hypertension and a low nephron number was first reported in 2003 [12]. The study analyzed autopsy kidneys from German patients who died in accidents. A comparison study of 10 age-matched hypertensive and normotensive subjects reported a total nephron number in the hypertensive group of approximately half that of the control group. A subsequent study reported that the total nephron number was associated with hypertension in US Whites but not in US Blacks [22]. Our study of nephron numbers in Japanese autopsy kidneys showed that the number of nephrons was lower in the hypertensive group than in the normotensive group (420,000 vs. 660,000) [20]. These results suggest that the relationship between total nephron number and hypertension may not be a universal phenomenon across races.

It is well known that nephron number, salt intake, and blood pressure are closely related to each other. For example, in normal rats, blood pressure is hardly increased by excessive salt loading. In one-kidney nephrectomized rats, normal salt intake does not increase blood pressure, but hypertension develops under high salt loading. When more than 70% of the kidney is removed, the blood pressure increases even under normal salt intake and becomes increasingly elevated in response to salt loading [26]. Salt sensitivity is closely related to glomerular hypertension and accelerated glomerulosclerosis, resulting in a further reduction in the number of nephrons. The number of nephrons is further reduced, resulting in a vicious cycle in which salt-sensitive hypertension is further amplified [27]. Previous studies have suggested that there are other mechanisms underlying low nephron numbers that are potentially implicated in blood pressure abnormalities. It has been suggested that a low nephron number may be associated with an increased fluid volume, increased renal salt sensitivity, activation of the renin-angiotensin-aldosterone system, sympathetic activation, and abnormal vasoconstrictor reactivity [28].

### Nephron number and aging

The process of age-related glomerulosclerosis has long been a focus of attention. Aging causes a reduction and subsequent loss of glomerular capillaries, and the afferent and efferent arterioles form a direct connection at this time, which maintains the blood flow in the medulla [29]. This, in combination with the arteriolar stiffness present in the cortex, leads to a reduction in cortical blood flow. This situation may contribute to the cortical predominance of renal parenchymal atrophy. In fact, the proportion of sclerotic glomeruli is reported to be increased in elderly individuals [30].

Several studies have assessed age-related changes in nephron number in the human kidney. Brenner et al. postulated that a reduction in the number of nephrons to < 500,000 per kidney at birth may be essential for the development of hypertension in adulthood [28]. Recently, there have been 2 such studies conducted: a stereological analysis series of 319 autopsy kidneys by Monash University and a CT angiography/biopsy analysis of 1,388 kidney donors by the Mayo Clinic [31, 32]. In the Monash data, the sharp decline in nephron number after 40 years old was not as pronounced as that in the Mayo Clinic data. Furthermore, the decline in nephron number after 70 years of age was not as marked as in the Mayo Clinic data. Of note, there were few kidneys from donors over 65 years old in the Monash series. A substantial age-related decline in nephron number was seen in the donor kidneys of the Mayo Clinic, as they were predominantly elderly with no elevated blood pressure or mild hypertension, which may explain the significant age-related decline in nephron number. Whether aging kidneys with an inherently low nephron number develop a more rapid decline in nephron number is an important topic for a future study.

### Nephron number and the development of CKD

**Fig. 2:**
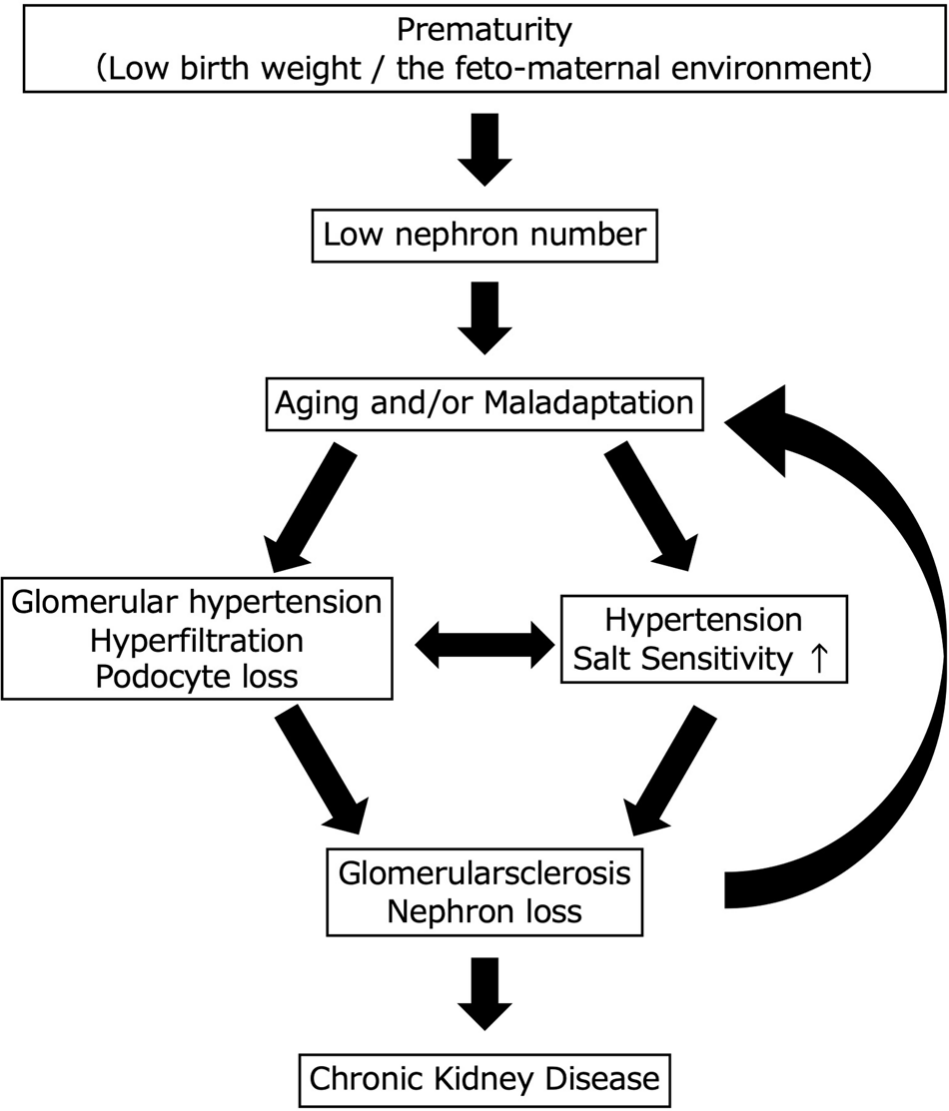
Vicious cycle involving nephron/podocyte loss, hypertension and chronic kidney disease. The decrease in nephron number in humans is influenced by the immaturity of kidney development due to the fetal-maternal environment and aging changes and maladaptation after birth. Podocyte loss and increased salt sensitivity lead to further nephron loss, resulting in a vicious cycle of reduced nephron numbers, hypertension, and chronic kidney disease

A congenital or acquired reduction in the number of nephrons leads to glomerular hyperfiltration and glomerular hypertension as well as glomerular injury, resulting in glomerulosclerosis and a further reduction in the number of nephrons (hyperfiltration theory). This vicious cycle is considered an important pathogenesis of CKD progression, regardless of the underlying disease [33] (Fig. 2). We also calculated the single-nephron glomerular filtration rate (SNGFR), a value obtained by dividing the estimated glomerular filtration rate (eGFR) by the number of nonsclerotic glomeruli, and examined its utility as a marker of glomerular hyperfiltration. Our analysis showed that the SNGFR was elevated only in the hypertensive group and not in the CKD group, although the degree of glomerular enlargement was similar between the groups. This finding suggests that the pathogenesis of hypertension is associated with glomerular hyperfiltration, whereas in the pathogenesis of CKD, there may be a disruption of compensatory mechanisms that is not reflected in the degree of glomerular enlargement [20]. Interestingly, we also estimated the nephron number and SNGFR in kidney transplant donors and found that the SNGFR was positively correlated with protein and salt intake, suggesting that dietary consumption is also an important determinant of the SNGFR [34].

Low nephron numbers at birth and the subsequent effects of obesity, diabetes, and hypertension accelerate the decline in nephron number through glomerular hyperfiltration, making CKD more likely to develop. The hyperfiltration theory, which is the concept that a reduction in nephrons and subsequent glomerular hyperfiltration as well as glomerular injury result in glomerulosclerosis and further nephron loss, is widely accepted. In response to this concern, researchers have focused on the number of podocytes, since depletion of injured podocytes is a hallmark of progressive kidney diseases associated with glomerulosclerosis [35–38]. In addition, our recent studies showed that the number of nephrons was significantly correlated with podocyte number in living donor kidneys and autopsy kidneys [39, 40]. These results suggest that nephron number and podocyte number are intrinsically well linked and may influence each other.

## The effect of CKD and hypertension on “podometrics”

### Podocyte endowment

Unlike nephron endowment, whether podocytogenesis and an increase in podocyte number are finished around the time of birth is unclear. Puelles et al. established a method for estimating podocyte number based on design-based stereology [41], which is the gold standard for estimating the number of targets of interest (e.g., the number of podocytes per glomerulus) within the kidneys. An autopsy study using design-based stereology reported that adults had a median of 558 podocytes per glomerulus, and children had a median of 452 podocytes per glomerulus, suggesting that podocyte numbers may increase during childhood [38]. Another autopsy study assessed fetal kidneys and found that the podocyte number per tuft was almost consistent from gestational weeks 22 to 39 [42]. The podocyte number in rats did not increase from the capillary-loop stage of glomerular development to 24 weeks old [43]. Whether the number of podocytes increases after birth seems to still be controversial, as no study has followed the same individuals with an adequate sample size in a longitudinal manner.

Another fundamental question is what factors regulate podocyte endowment. Some of the factors associated with nephron endowment were examined for podocyte endowment in experimental models. Maternal mild hypoxia in mice resulted in reduced podocyte numbers in male offspring but not in female offspring [44]. We recently reported that a maternal low-protein diet gives rise to offspring with a low podocyte endowment as well as low nephron endowment [45]. In a recent human study, we found that nonsclerotic nephron number was directly correlated with podocyte number per glomerulus in 50 autopsy kidneys from Japanese individuals without apparent kidney diseases [40]. These results suggested that the maternal environment is an important determinant of podocyte endowment, indicating that nephron and podocyte endowment either overlap or may be linked.

### Podocyte depletion hypothesis

**Fig. 3:**
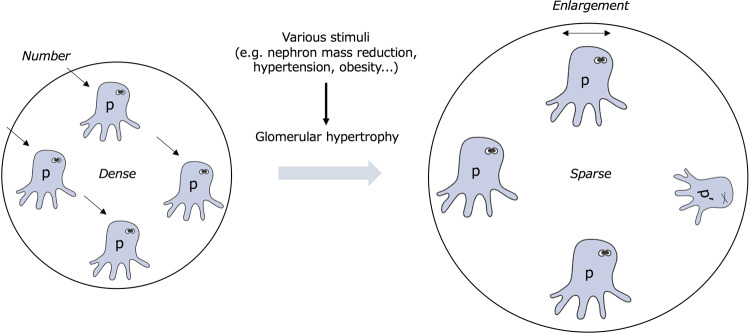
The concept of podometrics. In cases of glomerular hypertrophy due to a reduced total nephron mass, podocytes adapt by enlarging their bodies to maintain glomerular structure and filtration function. Podometrics evaluates the number, density, and volume of the “remaining” podocytes in situ in healthy and diseased glomeruli. p Podocyte, p’ Injured podocyte

Podocytes have a limited capacity to regenerate under normal conditions [46, 47]. Since the 2000s, much attention has been given to podocyte numbers and density. A landmark study using podocyte-specific diphtheria toxin receptor-expressing rats showed that podocyte injury itself causes glomerulosclerosis and proteinuria [36]. In addition, the degree of podocyte depletion was closely associated with glomerular pathological lesions, proteinuria, and kidney function. Podocyte hypertrophy (increased podocyte volume) can occur in response to various stimuli. This process is also considered to function as a method of compensation to maintain the coverage area of the glomerular capillaries under conditions of podocyte depletion [48, 49]. Fukuda et al. observed that the glomerular tuft volume exponentially increased with body weight gain in nephrectomized rats. In contrast, the total podocyte volume did not increase in proportion to glomerular volume [37]. Decompensatory hypertrophy and subsequent depletion of podocytes are important factors for the development and progression of glomerulosclerosis and proteinuria. Thus, podometrics, including the podocyte number, density, and volume, are important indices involved in the development and progression of CKD (Fig. 3). Wiggins proposed the podocyte depletion hypothesis, wherein effective podocyte loss and decompensated podocyte hypertrophy contributed to glomerulosclerosis and persistent proteinuria, leading to ESKD, irrespective of the cause of glomerular injury [50]. Since podocytes are intricately distributed over the surface of the glomerular capillary, the podocyte number and volume have not been estimated. Recently, however, several methods for estimating podometrics have been developed [39, 41, 51, 52]. These technical advances have provided growing evidence supporting the importance of podometrics in kidney health and diseases.

### Hypertension and podometrics

Hypertension contributes to podocyte injury and affects podometrics. The podocyte number per glomerulus in 41 patients with biopsy-proven hypertensive nephrosclerosis was lower than that in living kidney donors [53]. In addition, the urinary mRNA levels of podocyte markers from patients with hypertensive nephrosclerosis were higher than those in a control group. Naik and Wiggins et al. showed that the levels of urinary markers of podocytes in living kidney donors were directly correlated with the mean blood pressure within the normal range, indicating that accelerated podocyte loss can occur in hypertensive subjects prior to the development of apparent kidney disease [54]. An autopsy study by Puelles et al. using a design-based strategy method showed that hypertension was associated with a lower podocyte density but not a lower podocyte number per tuft in 19 Caucasian Americans without apparent kidney diseases [55]. Our podometric study assessed living donor kidneys and found that hypertension was associated with a lower podocyte density but not a lower podocyte number per tuft [39]. In addition, hypertension was associated with a larger podocyte volume, independent of age or glomerular volume, in the first report showing that a hypertensive status was associated with an increased podocyte volume [39]. This finding suggested a possible link between podocyte hypertrophy and factors involved in the pathogenesis of hypertension, such as angiotensin II or mammalian target of rapamycin (mTOR) [56, 57]. However, the causal relationships between the podometric changes and hypertension are currently unknown, since these findings are all based on clinical observational studies. Further interventional studies are needed to resolve this issue.

### Other factors associated with podometrics

Normal aging is the most powerful determinant of podocyte depletion during life. Puelles et al. showed that aging was associated with both a lower podocyte density and a lower podocyte number per tuft in autopsy kidneys from 19 Caucasian Americans without apparent kidney diseases using design-based stereology [55]. A study that assessed 89 kidneys from kidney donors and patients with kidney tumors using model-based stereology showed that the podocyte number and density linearly decreased with age [49]. Furthermore, the urinary mRNA levels of podocyte-associated components were found to be increased in older subjects (≥ 60 years old) compared with younger subjects [49]. Our study simultaneously measured the nephron number and podometrics in 30 Japanese living-donor kidneys and revealed that the total number of podocytes per kidney (the product of the podocyte number per tuft and the number of nonsclerotic glomeruli) declined with age at a rate of 5.63 million podocytes per kidney per year, which equates to a loss of 643 podocytes per kidney per hour from healthy kidneys [39].

Podocyte depletion has been reported in most glomerular diseases, including diabetic nephropathy [58–61], IgA nephropathy [62], obesity-related glomerulopathy [63], and Alport syndrome [64]. Since most of these studies are cross-sectional, the association between podocyte depletion and subsequent kidney outcomes remains to be defined.

With regard to racial differences in podometrics, we found no apparent differences in podocyte numerical indices in previously reported data from Caucasians and our data from Japanese living-kidney donors [39]. These studies involved moderately heterogeneous cohorts, including living and deceased donor kidneys and nephrectomy samples, and the analyses used different methods for estimating podometrics. Given that our previous study showed fewer nephrons in Japanese subjects than in other races, further investigations simultaneously assessing the nephron number and podometrics to compare racial differences are warranted.

## Strategic antihypertensive therapy to protect nephrons and podocytes

### Target blood pressure in patients with CKD

The ultimate goal of blood pressure management in CKD patients with hypertension is to prevent progression to ESKD and the development of cardiovascular disease (CVD). The Japan Society of Hypertension Guidelines for the Management of Hypertension (JSH 2019) recommend an optimal target blood pressure of < 130/80 mmHg for hypertensive CKD patients with persistent proteinuria and < 140/90 mmHg for hypertensive CKD without proteinuria [65].

There are some caveats regarding antihypertensive treatment for CKD patients. First, the disappearance of diurnal variation in blood pressure should be considered in CKD patients. Clinically, loss of nocturnal blood pressure decrease is frequently observed in CKD patients, presumably due to altered salt handling of the kidney [66]. It is therefore important to control 24-h blood pressure in patients with CKD. Second, proteinuria levels should be carefully monitored as a surrogate for renal impairment, since time-dependent and time-averaged levels of urinary protein excretion during follow-up have been shown to correlate well with the subsequent development of ESKD and CVD [7, 8]. Experimental studies using nephrectomy models have shown that further aggressive antihypertensive therapy avoids the gradual glomerulosclerosis seen in the absence of overt hypertension [67]. Thus, in patients with CKD, the optimal blood pressure should be individualized, even in the absence of hypertension criteria, with a particular emphasis on proteinuria levels as a surrogate indicator of renal and cardiac prognosis. Third, some CKD patients may require special caution with regard to excessive antihypertensive treatment. In cases of advanced systemic atherosclerosis, including renal arteries, antihypertensive treatment may cause a rapid decline in renal function. This decline occurs because patients with advanced nephrosclerosis have lost autoregulatory function, and antihypertensive treatment directly leads to a decrease in renal plasma flow. Therefore, antihypertensive therapy for CKD patients, typically elderly CKD patients, with plausible advanced nephrosclerosis should be administered with caution. Given these subgroups of patients, JSH 2019 recommends that the target blood pressure be set at < 140/90 mmHg in CKD patients without proteinuria.

### Antihypertensive therapy for CKD patients

A number of experimental studies have shown beneficial effects of angiotensin-converting enzyme (ACE) inhibitors in various models of advanced kidney disease, including 5/6 nephrectomy models with extensive removal of nephron mass. The beneficial effects of ACE inhibitors have been attributed primarily to a reduction in single-nephron glomerular hypertension [68, 69], and morphological changes in podocytes correlate with glomerular vulnerability and are restored by therapy with ACE inhibitors [35]. Subsequent clinical trials have consistently demonstrated the renoprotective effects of renin-angiotensin aldosterone system (RAAS) inhibitors, including angiotensin type-1 receptor blockers (ARBs) or ACE inhibitors, in diabetic and nondiabetic CKD patients with persistent proteinuria [70, 71]. Based on these findings, RAAS inhibitors have been recommended as first-line drugs to protect against loss of nephrons and podocytes in CKD patients with hypertension, especially those who present with persistent proteinuria.

Several prospective controlled trials have demonstrated that diuretics, including thiazide diuretics [72, 73] and mineralocorticoid receptor (MR) antagonists [74, 75], reduce proteinuria levels in patients with CKD. The anti-proteinuric effect of thiazide diuretics has been shown to be enhanced by a low-salt diet or in combination with RAAS inhibitor therapy [72, 73]. Experiments using nephrectomy models have shown that both thiazide diuretics [72] and MR antagonists [76] have renoprotective and cardioprotective effects. It has been demonstrated that MR is expressed in glomerular podocytes, and the antiproteinuric effect of MR antagonists can be exerted via reduction of podocyte injury [77]. Furthermore, MR activation is closely associated with multidrug-resistant hypertension, which is often observed in CKD patients [78]. Based on these findings demonstrating the renoprotective effects of thiazide diuretics and MR antagonists, these drugs may be applicable for patients with proteinuric CKD. Note that diuretics are often associated with clinically important adverse effects, especially when administering MR antagonists to patients with CKD, and they should be used with caution for hyperkalemia.

In addition to their hypoglycemic effects, renoprotective effects of sodium glucose cotransporter-2 (SGLT-2) inhibitors have also been suggested [79, 80]. The mechanisms involved in the anti-proteinuric effects of SGLT-2 inhibitors are not fully understood but essentially involve improving single-nephron hyperfiltration and reducing salt over-reabsorption from the proximal tubules [81]. SGLT-2 inhibitors are known to have antihypertensive effects, which may be additionally related to the reduction in proteinuria levels [82, 83]. The majority of patients included in clinical trials examining the renoprotective effects of SGLT-2 inhibitors are those already receiving combination therapy with RAAS inhibitors [79, 80]. The results may suggest additive and synergistic effects of SGLT-2 inhibitors at the single-nephron level that are distinct from the effects of RAAS inhibitors. Importantly, the renoprotective effects of SGLT-2 inhibitors have been demonstrated in patients with advanced CKD stages who are likely to have a substantial reduction in the numbers of nephrons and podocytes. Several experimental studies examining the effects of SGLT-2 inhibitors using nephrectomy models are consistent with these findings [84].

A series of studies on the renoprotective effects of antihypertensive drugs revealed the multifaceted nature of CKD progression at the single-nephron level. Modifications in salt handling are commonly implicated in the effects of these drugs. This information suggests that salt sensitivity mechanisms are intrinsically involved in the progression of proteinuric CKD. It is noteworthy that all nephroprotective antihypertensive drugs show an initial GFR decline following their administration [85, 86]. Careful evaluation and monitoring are warranted after the administration of these drugs, as the GFR decline may be enhanced in patients with advanced CKD with a low nephron mass who are already undergoing antihypertensive therapy with other renoprotective drugs.

### Lifestyle modifications

Early studies have suggested that a reduced filtration surface is one of the mechanisms underlying impaired renal salt handling associated with congenital or acquired nephron mass reduction, leading to salt-sensitive hypertension [87, 88]. The appearance of salt intake-dependent proteinuria has been shown to increase in frequency with the progression of the CKD stage [89]. In addition, the antiproteinuric effects of RAAS inhibitors have been found to be enhanced in salt-restricted patients compared with those who are not salt-restricted [90]. These findings suggest that salt restriction is a particularly important component of effective antihypertensive therapy, especially in patients with advanced CKD. Currently, the guideline-based salt intake recommendation for hypertensive patients is uniformly defined as ≤ 6 g per day, irrespective of CKD [65]. The evidence on the recommended salt intake in relation to hypertensive disorders has been established by population-based studies, so the optimal salt intake is not individualized according to CKD stage or urinary protein excretion level [91].

Recent studies have shown the importance of an appropriate quantity of protein intake from different food sources for preventing incident hypertension [92]. In patients with advanced CKD, however, a high protein intake may promote CKD progression and the accumulation of uremic toxins [93]. Note that excessive protein restriction may otherwise promote frailty due to muscle weakness and reduce the quality of life in elderly patients. Therefore, the appropriate protein intake for CKD patients should be determined on an individual basis, carefully taking into account each patient’s clinical background.

Obesity accelerates kidney injury by several mechanisms that may or may not be dependent on hypertension, including those induced by adipocytokines released from visceral fat [94]. Obesity has been identified as one of the strongest clinical risk factors for the appearance of proteinuria in patients with reduced nephron mass, such as kidney transplant donors and patents with congenital nephron deficit [95, 96]. Obesity has been consistently identified as a factor inducing podocyte injury with proteinuria secondary to glomerular hypertrophy [90]. Based on these findings, obesity is hypothesized to be a typical condition inducing renal injury via mechanisms associated with a “relative” reduction in nephrons and podocytes. The mismatch between body size-nephrons and glomerular size-podocytes is currently considered a relevant factor involved in obesity-related kidney injury [63, 97]. Since obesity increases salt retention, induces volume overload, and can cause obesity-related hypertension, weight loss is considered a high-priority viable approach for managing obese CKD patients [97].

### Approaches for elderly patients with advanced glomerulosclerosis

The kidneys of elderly individuals are characterized by advanced atherosclerosis and decreased autoregulatory capacity of the intrarenal arteries. This situation may predispose elderly hypertensive patients to further pressure overload injury to the kidneys. Thus, it is intrinsically important to reduce pressure overload in elderly hypertensive patients. In support of this idea, a recent large-scale trial showed that strict blood pressure reduction was beneficial for reducing the risk of developing CVD in elderly hypertensive patients [98]. However, aging has a significant impact on the development of nephrosclerosis, which accompanies a decreased number of nephrons and podocytes, creating a unique situation that may influence the response to salt restriction and antihypertensive drug therapies [99]. In addition, elderly kidneys may be more sensitive to an impaired GFR than kidneys in younger individuals due to a decreased renal blood flow following blood pressure reduction. Furthermore, the elderly may be more prone than the young to side effects associated with antihypertensive drug therapy, such as syncope, injurious falls, and electrolyte abnormalities [65]. Therefore, the target blood pressure for elderly CKD patients with hypertension should be determined by balancing the harms and benefits.

### Perspectives in Asia

The Japanese population has long been known to be at high risk of hypertension and hypertensive disorders such as stroke [100]. In recent years, the incidence of hypertension has been increasing despite a downward trend in salt intake among the Japanese [65]. Thus, the increasing incidence of hypertension in Japan may be due in part to other factors associated with hypertension, such as aging of the overall population and increased incidences of obesity and metabolic syndrome due to the westernization of the Japanese diet [65]. Autopsy studies have shown that the Japanese tend to have fewer nephrons than other races [20]. The presence of fewer nephrons with a possible reduced renal salt handling capacity in Japanese individuals may be associated with greater susceptibility to hypertension than in other races. A similar scenario can be assumed for other Asian countries where the majority of the population shares a similar race to Japan. Thus, basic studies investigating the number of nephrons and podocytes in the Asian population may be required to address this issue. However, counting nephrons and podocytes using autopsy kidneys is time- and labor-intensive, and an important commonality among current clinical approaches is the reliance on biopsy materials, which makes these procedures inherently invasive [101]. Therefore, a noninvasive and simple method of investigating the number of nephrons and podocytes at the population level is needed. Such methods may be applicable to larger numbers of individuals, enabling a better assessment of patients at high risk of conditions such as hypertension and CKD, including those in Asian countries.

In recent years, unfavorable social trends associated with kidney maturation, such as underweight in pregnant women and low birth weight in infants, have become more prominent worldwide, especially in Asian countries [2]. In addition, given the social context of increased obesity and superaging, there is a strong concern that the number of nephrons and podocytes has been declining in recent years, especially in the Asian population. Therefore, to reduce the risk of CKD progression and CVD in Asian countries, there is a need to establish social education programs that stress the importance of maintaining a good pregnancy environment and dietary habits in parallel with appropriate blood pressure control [102].

## Conclusions

The interrelationship between reduced nephron numbers, hypertension, and CKD is consistent with the concept of common pathways of renal disease progression established over previous decades in the field of nephrology. Recent studies have suggested that the number and function of podocytes constitute an important part of this relationship. A reduced nephron number with fewer filtration surfaces and an impaired salt handling capacity may induce single-nephron hyperfiltration, leading to maladaptive podocyte injury and persistent proteinuria. To interrupt this vicious cycle of CKD progression, the combination of lifestyle modification and appropriate antihypertensive drug therapy is essential, with full consideration of each individual’s clinical background. There is substantial concern that the number of nephrons and podocytes in the Asian population has been declining in recent years. Therefore, multifaceted measures, including sociological approaches, are urgently needed to prevent the incidence of hypertension and CKD in Asian countries.
